# Accuracy of the Imageless Mode of the ROSA Robotic System for Targeted Resection Thickness in Total Knee Arthroplasty: A Prospective, Single Surgeon Case‐Series Study

**DOI:** 10.1002/rcs.70029

**Published:** 2024-12-23

**Authors:** Zakareya Gamie, Eustathios Kenanidis, Georgios Douvlis, Nikolaos Milonakis, Alexander Maslaris, Eleftherios Tsiridis

**Affiliations:** ^1^ Tsiridis Orthopaedic Institute ICAROS Clinic Thessaloniki Greece; ^2^ Centre of Orthopaedic and Regenerative Medicine (CORE) Center for Interdisciplinary Research and Innovation (CIRI) Aristotle University of Thessaloniki (AUTH), Balkan Center Thessaloniki Greece; ^3^ Academic Orthopaedic Department Aristotle University Medical School, General Hospital Papageorgiou Thessaloniki Greece; ^4^ Nuffield Department of Orthopaedics, Rheumatology and Musculoskeletal Sciences (NDORMS) Oxford University Hospital NHS Oxford UK

**Keywords:** arthroplasty, knee, robotics, technology

## Abstract

**Background:**

We investigated the accuracy of targeted resection thickness in patients undergoing primary Total Knee Arthroplasty (TKA) using the ROSA robotic system.

**Methods:**

Calliper measurements of the distal femur (DF), proximal tibia (PT), and posterior condyles (PC) were taken in 44 patients from June 2023 to January 2024.

**Results:**

Planned and actual resection depth difference was 0.67 mm ± 0.6 mm (mean ± SD) (*p* = 0.217) and 0.94 mm ± 1.15 mm (*p* = 0.4) for medial and lateral DF, 0.93 mm ± 0.81 mm (*p* = 0.001) and 0.89 mm ± 0.8 mm (*p* = 0.008) for medial and lateral PT, and 1.1 mm ± 0.97 mm (*p* = 0.001) and 1.04 mm ± 0.79 mm (*p* = 0.001) for medial and lateral PC, respectively.

**Conclusion:**

The ROSA robotic system can achieve a high degree of accuracy for planned resection thickness. Results are valid only for the imageless ROSA TKA in patients with primary knee osteoarthritis.

## 1 Introduction

Robotically assisted Total Knee Arthroplasty (RaTKA) is gaining popularity as it enables a more precise and accurate bone resection strategy, eliminates errors and provides a personalised approach to TKA. However, the evidence for each robotic system in RaTKA varies in quality and quantity, requiring individual assessment based on specificity [1]. Robotic systems can improve resection levels and angles [2] and reduce resection thickness compared with navigation [3]. The ROSA Knee System (Zimmer Biomet, Warsaw, IN) is a semi‐active robotic system that allows the surgeon to place the cutting guide in the planned position and resect the bone through the cutting guide [4, 5]. It is different from other systems such as the MAKO robotic arm‐assisted technology (MAKO, Stryker, USA), which has a haptic arm keeping the surgeon within restricted boundaries [6], or the NAVIO or CORI robotic‐assisted milling system (Smith & Nephew), which is passive and retracts when reaching a boundary [7]. Active systems also exist in which the surgeon is not involved in the process [8, 9]. Besides, varying preoperative planning methods exist in different robotic systems. The ROSA Knee System can be used in either image‐based mode based on pre‐operative 2D calibrated radiographs or imageless mode based on intra‐operative bony registration. On the other hand, the MAKO knee system is an image‐based system based on a pre‐operative Computerised Tomography (CT) scan. Image‐based and imageless systems can have similar accuracy in terms of coronal alignment, but the imageless system can have better accuracy for sagittal alignment [10].

As a result, errors in bone cutting that can occur must be explained separately for each robot. The ROSA Knee System allows for the validation of cuts, with the surgeon able to perform a recut if necessary. The accuracy of the resection depth has been quoted as 0.7 mm or less for the ROSA system in cadaveric studies [5, 11]. Limitations of a cadaveric study include normally aligned limbs with less osteoarthritis when compared to clinical cases where the bone quality can vary (osteoporotic and hard subchondral bone) as well as the degrees of deformity, which can also exist with different ligament tensions. Another cadaveric study using the Stryker MAKO robotic arm‐assisted technology for TKA, compared the position of trials relative to the plan without information on planned versus achieved resection thickness [12]. Errors can range from 0.6 to 2.4 mm for both femoral and tibial implants in early studies of robotic assistance using the burr [13]. A recent patient study using the ROSA system analysed the accuracy of the angle of the cut produced using three‐dimensional measurements from 3D CT of the whole leg at 2 weeks postoperative TKA [14]. It was reported to be within 1°, which supported the findings of previous cadaveric and patient studies [14]. Therefore, investigation of the degree of accuracy of the angle of the cut by the ROSA Knee System has been carried out in both cadaveric studies and patients; however, the accuracy of resection depth has yet to be analysed in patients using imageless and image‐based systems. The main aim of this study was to analyse the planned resection depth measurements and compare this with actual calliper measurements, accuracy between observers, to assess percentage of resections below 1 mm and patient factors that can influence the accuracy in a group of patients undergoing imageless robotic‐assisted TKA using the ROSA Knee System from a senior surgeon.

## 2 Methods

### 2.1 Selection of Patients

Forty‐four patients with symptomatic end‐stage knee osteoarthritis underwent primary RaTKA at the same single centre between September 2023 and January 2024. A senior surgeon trained in RaTKA performed all the procedures. The NexGen Complete Knee Solution Legacy Knee Posterior Stabilized (LPS Flex precoat) (Nexgen Legacy, Zimmer Biomet, Warsaw, IN) was used in all procedures that were performed with the aid of the imageless mode of the ROSA TKA robotic system (Zimmer Biomet). Patients with unilateral knee osteoarthritis undergoing primary TKA were recruited for the study. Patients suffering from neurological disorders, inflammatory arthropathies, or any other secondary arthritis were excluded from the study. IRB approval was obtained for the study from the faculty of health sciences, medical department, Bioethics and Ethics committee, Aristotle University, Thessaloniki, Greece (IRB Number: 4/13.02.2024). A total of 44 patients underwent ROSA RaTKA between July 6, 2023 and January 11, 2024 by a single senior surgeon. All patients fulfiled the inclusion criteria and were included in the study. The age of the patients was 69.6 ± 11.8 years (mean ± SD). Seventeen patients were male, and 27 were female; 27 right TKAs and 17 left TKAs were performed. The BMI of the patients was 28.9 ± 2.6 kg/m2 (mean ± SD). Forty varus knees and 4 valgus knees were operated on. The pre‐operative tibiofemoral angle (TFA) of the varus knee group was 4.43 ± 4.130 and the TFA angle of the valgus knee group was 11.1 ± 4.190 (mean ± SD).

### 2.2 Surgical Technique

Fully cemented posterior stabilised TKAs were performed in all patients. Thigh tourniquet pressure was applied prior to skin incision and maintained until skin closure. A deep drain was used for the first day after surgery. The aim was to restore constitutional bony alignment and balance the laxity of the soft tissues by placing and sizing implants in a manner that respects variations in individual anatomy and soft tissues within defined limits. To achieve balanced flexion‐extension gaps and equal mediolateral soft tissue tension, the surgeon first performed the necessary soft tissue releases and then the bone resections that were adjusted within the limits of  ± 5° varus‐valgus cuts of the ROSA Knee System.

**FIGURE 1:**
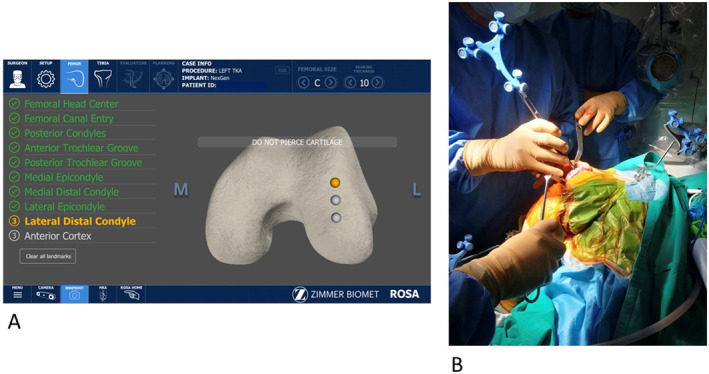
A—Femoral registration landmarks required by the ROSA Knee System. B—Intraoperative image of the use of the probe on the medial distal femur to register this landmark into the ROSA Knee System and not pierce the cartilage.

More specifically, following the arthrotomy, the appropriate soft tissue releases to the mid‐coronal tibial plane and osteophyte removal were performed. Then, the standard tibial and femoral landmarks were registered based on the ROSA protocol. During the procedure, the aim was not to pierce the cartilage (or expose bone) when registering the landmarks (Figure 1). Then, the surgeon performed the intraoperative planning using the specialised software. Planning of the resection depths was made following a functional alignment strategy to achieve a balanced knee in extension and flexion by accurately addressing a combination of factors, such as the placement and sizing of implants, so that it respects individual variation in anatomy and soft tissue laxity [15, 16, 17]. The robot's arm with the universal cutting jig was then positioned close to the patient's leg precisely according to the surgical plan. The surgeon then secured the jig to the bone using pins, and the necessary resections were performed. Following the distal femur and proximal tibia cuts, validation was carried out using the verification tool to measure and confirm the resection level and angle. The ROSA Knee System was used to evaluate the extension gap with a static spacer in ‘evaluation‘ mode. Additional ligament and soft tissue releases were made as required to achieve proper balance. Using the planning system and the manual ‘pulling’ manoeuvre of the femur in 90° knee flexion, the flexion gap and femoral component rotation were evaluated, including the resection level of the posterior femoral condyle. After intraoperative evaluation of the gap produced, laxity, and determination of femoral component rotation, the ROSA arm was moved to the bone in the ‘collaborative’ mode and pins placed through the four in one cut guide. This was followed by the placement of the jig and two pins to secure it in place. Cuts to the following regions were made through the cutting block: lateral and medial posterior femoral condyle, ventral and oblique trochlea, and anterior and posterior chamfer cut. If a recut was planned and undertaken, the recut bone (distal femur and proximal tibia) was also measured and recorded with the associated planned and validated measurements. All the cuts and the verification of the cuts were carried out by one surgeon. Trial implants were inserted, and the leg axis was assessed. Standard instrumentation was then used to prepare the tibial metaphysis, and the final implants were implanted.

### 2.3 Cuts and Calliper Measurements

**FIGURE 2:**
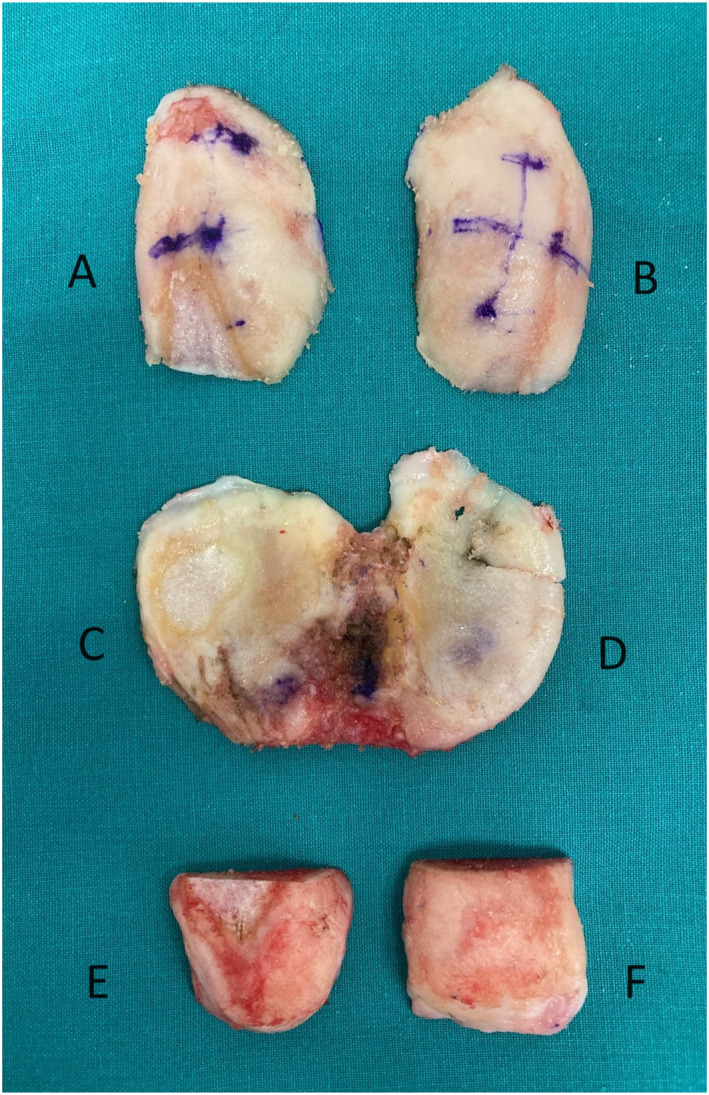
The resected specimens produced intraoperatively: A—lateral distal femur, B—medial distal femur, C—lateral tibial plateau, D—medial tibial plateau, E—lateral posterior femoral condyle, and F—medial posterior femoral condyle.

**FIGURE 3:**
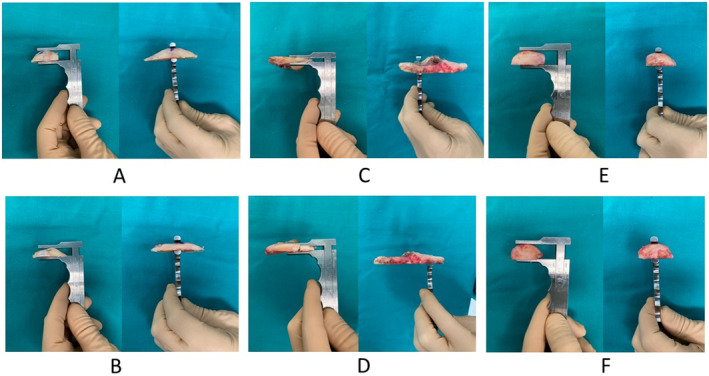
Calliper measurement method of the resected specimens. A—lateral distal femur = 8.5 mm, B—medial distal femur = 11 mm, C—lateral tibial plateau = 2 mm, D—medial tibial plateau = 3.5 mm, E—lateral posterior condyle = 12.5 mm, and F—medial posterior femoral condyle = 13 mm.

As part of the surgical procedure, the following regions were resected for analysis: A—lateral distal femur, B—medial distal femur, C—lateral tibial plateau, D—medial tibial plateau, E—lateral posterior femoral condyle, and F—medial posterior femoral condyle. The aim was to take measurements from specific regions of the resected bone cuts, which also corresponded to the regions taken for registration into the ROSA Knee System (Figure 2). The thickness of the resected bones was evaluated using a calliper. Calliper measurements were made of the resected specimens two times and by two different observers (ZG and EK) independent of the surgery (Figure 3). Statistical analysis was performed to assess for interobserver agreement and for any significant differences between the planned and calliper measurements. When the two observers had different measurements, their average was considered valid. The calliper measurements were then compared to the intraoperative planned resection amounts to confirm RaTKA accuracy.

### 2.4 Statistical Analysis

To assess our study's necessary sample size, we based this on the standard deviation of measurements reported by Paratte et al. 2019 [5], which ranged from 0.6 to 1.1 mm. To detect a difference of 0.3 mm in our study with a standard deviation of 0.8 mm, a two‐sided alpha of 0.05%, and 90% power, at least 37 patients were required for the study. We checked for normality using the Kolmogorov–Smirnov or Shapiro–Wilk test. *p* value < 0.05 was considered statistically significant. Standard statistical procedures were used to calculate descriptive statistics. Statistical tests were two‐tailed. To compare the planned and calliper measurements, we used the paired *t*‐test or Wilcoxon signed‐rank test. The intra‐class correlation coefficient was used to correlate the measurements between the observers. Multivariate linear regression analysis was used to explain the amount of variance of the difference in resection cut measurements that can be explained by clinical and radiological parameters. The statistical analysis was conducted using IBM SPSS software (version 27.0).

## 3 Results

**FIGURE 4:**
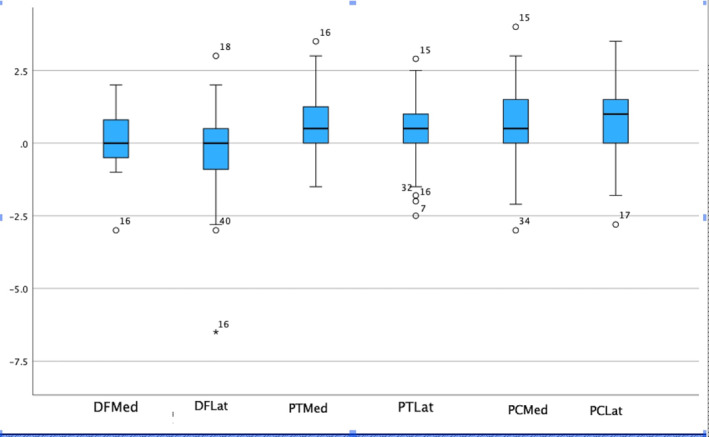
Box plots including the maximum, minimum, median, interquartile range and outlier values for each of the bone resections. Positive values indicate that less bone was resected than planned. Distal femur (DF), proximal tibia (PT), posterior condyle (PC), Med: medial, Lat: lateral.

Intra‐class correlation coefficient (ICC) between 0.75 and 0.9 (good reliability) was observed in four measurements and between 0.9 and 1.0 (excellent reliability) in the other two measurements of bony resections between the observers (Table 1), according to criteria by Landis and Koch [18]. The range of values for each resected knee part is presented in Figure 4.

**TABLE 1:** Interobserver correlation coefficient comparing the observations by two different observers for each bone resection. Distal femur (DF), proximal tibia (PT), posterior condyle (PC).

	DF lateral	DF medial	PT lateral	PT medial	PC lateral	PC medial
ICC	0.89	0.86	0.86	0.70	0.95	0.98

The mean difference (mean ± SD) between the planned resection and the actual calliper measurements were found to be 0.67 ± 0.6 and 0.94 ± 1.15 for the medial and lateral distal femoral resections, 0.93 ± 0.81 and 0.89 ± 0.8 for the medial and lateral proximal tibial resections, and 1.1 ± 0.97 and 1.04 ± 0.79 for the medial and lateral posterior femoral condyles resection, respectively (Table 2). The mean difference between the planned and the measured calliper values was not significantly different for the medial and lateral distal femoral resections; however, the mean difference between the planned and the measured calliper values was significantly different for the medial and lateral proximal tibial and posterior femoral condyles resections for all cases and for varus knees (Tables 2 and 3a). Only 4 valgus cases were performed and analysed, and no significant difference was found between the planned and measured resection values (Table 3b).

**TABLE 2:** Comparison between the mean planned and actual bone resection of group cases.

Bone resection^a^	Mean planned (mm)^a^	Mean actual (mm)^a^	Mean difference (mm)^a^	*p*	Max error (mm)	≤ 1 mm (%)
**Distal femur**						
Medial	10.96 (2.14)	10.79 (2.34)	0.67 (0.6)	0.217^b^	3.0	84.1
Lateral	9.26 (1.77)	9.46 (1.95)	0.94 (1.15)	0.400^b^	6.5	77.3
**Proximal tibia**						
Medial	4.16 (1.63)	3.45 (1.41)	0.93 (0.81)	< 0.001^b^	3.5	72.7
Lateral	9.02 (1.74)	8.55 (1.91)	0.89 (0.80)	0.008^b^	2.9	68.2
**Posterior femoral condyles**						
Medial	14.27 (1.60)	13.59 (1.77)	1.1 (0.97)	0.001^b^	4.0	56.8
Lateral	11.10 (1.50)	10.40 (1.96)	1.04 (0.79)	< 0.001^b^	3.5	65.9

^a^ The values are given as the mean with the standard deviation (±) in parentheses.

^b^ Tests performed using paired samples *t*‐test.

**TABLE 3a:** Comparison between the mean planned and actual bone resection of group cases in varus knees.

Bone resection^a^	Mean planned (mm)^a^	Mean actual (mm)^a^	Mean difference (mm)^a^	*p*	Max error (mm)	≤ 1 mm (%)
**Distal femur**						
Medial	11.28 (1.45)	11.12 (1.87)	0.66 (0.62)	0.255^b^	3.0	85
Lateral	9.4 (1.79)	9.53 (1.99)	0.89 (1.16)	0.603^b^	6.5	77.5
**Proximal tibia**						
Medial	4.01 (1.38)	3.36 (1.33)	0.87 (0.81)	< 0.001^b^	3.5	72.5
Lateral	9.3 (1.33)	8.84 (1.54)	0.44 (1.15)	0.016^b^	2.9	67.5
**Posterior femoral condyles**						
Medial	14.36 (1.6)	13.61 (1.79)	0.76 (1.33)	< 0.001^b^	4.0	52.5
Lateral	11.15 (1.53)	10.40 (2.01)	0.74 (1.13)	< 0.001^b^	3.5	62.5

^a^ The values are given as the mean with the standard deviation (±) in parentheses.

^b^ Tests performed using paired samples *t*‐test.

**TABLE 3b:** Comparison between the mean planned and actual bone resection of group cases of valgus knees.

Bone resection^a^	Mean planned (mm)^a^	Mean actual (mm)^a^	Mean difference (mm)^a^	*p*	Max error (mm)	≤ 1 mm (%)
**Distal femur**						
Medial	7.7 (4.79)	7.5 (4.18)	0.7 (0.4)	0.705^b^	1.3	75
Lateral	7.87 (0.85)	8.7 (1.5)	1.37 (1.1)	0.357^b^	3.0	75
**Proximal tibia**						
Medial	5.62 (3.25)	4.37 (2.09)	1.25 (1.1)	0.066^b^	3.0	75
Lateral	6.2 (2.94)	5.62 (3.03)	0.67 (0.69)	0.285^b^	1.5	75
**Posterior femoral condyles**						
Medial	13.25 (1.32)	13.37 (1.79)	0.12 (0.85)	0.785^b^	1.0	100
Lateral	10.62 (1.31)	10.37 (1.70)	0.25 (0.86)	0.577^b^	1.0	100

^a^ The values are given as the mean with the standard deviation (±) in parentheses.

^b^ Tests performed using paired samples Wilcoxon signed‐rank test.

The percentage of measurements with less than 1 mm difference between the planned and the actual calliper measurements was more favourable for the medial (84.1%) and lateral (77.3%) distal femoral resections. The percentage of measurements with less than 1 mm difference between the planned and the actual calliper measurements was 72.7% and 68.2% for the medial and lateral proximal tibial resections, and 56.8% and 65.9% for the medial and lateral posterior femoral condyles resection, respectively (Table 2).

Our regression analysis model, which included the age, sex, and TFA, explained the 0.6%–11% variance in the difference between the planned resection and the actual calliper measurements for all measurements (Table 4); however, none of the R square values were statistically significant. Of these measurements, only the TFA made a significant but non‐substantial contribution to the model concerning the lateral posterior condyle resection difference (Table 4).

**TABLE 4:** Linear logistic regression analysis: models explaining part of the difference of distal femur, proximal tibia and posterior condyle cuts between the planned and actual values with age, sex and tibiofemoral angle (TFA) values.

				Standardised coefficients	t	*p* value
Model		R (R square)	Significance	Beta		
Distal femoral	Age			0.000	0.039	0.969
Medial cut	TFA	0.079	0.969	−0.006	−0.262	0.794
Difference	sex	(0.006)		0.094	0.447	0.658
Distal femoral	Age	0.314	0.240	−0.017	−1.062	0.295
Lateral cut	TFA	(0.099)		0.19	0.479	0.634
Difference	Sex			0.726	1.904	0.064
Proximal tibial	Age	0.184	0.706	−0.003	−0.299	0.766
Medial cut	TFA	(0.034)		0.034	1.154	0.255
Difference	Sex			0.031	0.112	0.911
Proximal tibial	Age	0.118	0.903	0.001	0.061	0.952
Lateral cut	TFA	(0.014)		−0.014	−0.470	0.641
Difference	Sex			−0.134	−0.483	0.632
Posterior condyles	Age	0.283	0.336	0.011	0.849	0.401
Medial cut	TFA	(0.080)		−0.041	−1.207	0.235
Difference	Sex			−0.400	−1.231	0.225
Posterior condyles	Age	0.332	0.194	0.009	0.817	0.419
Lateral cut	TFA	(0.110)		−0.059	−2.165	0.036
Difference	Sex			0.145	0.560	0.579

## 4 Discussion

RaTKA is gaining popularity, enabling more accuracy and precision [19, 20] and adopting a more personalised alignment technique in the TKA [15, 16]. Previous cadaveric studies have found great accuracy of ROSA RaTKA in the resection depth [5, 11], which has been reported to be comparable to the findings of studies investigating navigation and patient‐specific instrumentation [5]. Our findings show that the imageless mode of ROSA RaTKA is highly accurate. The mean difference between the planned and the actual calliper measurements in our study ranged between 0.67 and 1.1 mm for distal femoral, proximal tibial and posterior condyles measurements.

Our results, as well as previous ones, demonstrated that the RaTKA may have different accuracy for various resected parts of the knee. In the cadaveric study by Parratte et al. in 2019, using the ROSA Knee System on 15 cadavers, 30 knees reported the distal medial femur cut accuracy compared to the planned at 0.4 mm ± 0.9 mm, distal lateral femur at 0.1 mm ± 0.9 mm, medial posterior femur at 0.2 mm ± 0.9 mm, lateral posterior femur at −0.1 mm ± 0.8 mm, the proximal medial tibial plateau at 0.7 mm ± 0.6 mm and proximal lateral tibial plateau at 0.2 mm ± 1 mm [5]. A further cadaveric study by Seidenstein et al. in 2021, also using the ROSA Knee System in seven cadavers and 14 knees, reported less accurate results without separate medial and lateral measurements with the distal femur cut accuracy compared to the planned at 0.7 mm ± 0.7 mm, posterior femur at 0.6 mm ± 0.5 mm and proximal tibia at 0.7 mm ± 0.6 mm [11]. We found values going down to 0 mm mean difference between the planned and actual calliper measurement for the different cuts but also differences in several millimetres. Medial tibial plateau and posterior femoral condyle resection demonstrated greater variability than the distal femoral resection cuts.

Although the measurements using ROSA RaTKA were very accurate, our study also showed that there may be a greater discrepancy between the planned and the actual resection cuts between the cadaveric and real patient studies. Reasons for inaccuracies and errors between the planned and actual bone resection measurements can include differences in bone density and quality. For example, when cutting hard sclerotic bone, bending of the saw blade can occur. In thin and weak osteoporotic bone, greater amounts of bone resection can potentially occur, as well as the movement of the cutting block, leading to errors. Tilting of the blade has also been reported as a concern, particularly for the cutting of the distal femur because of deviation due to the weight of the saw cutting in an extended position and additional adjustment by the surgeon [14]. However, our study did not find a relationship between age and sex that reflects the probability of osteoporosis as well as the TFA and the difference of resection between the planned and the actual calliper measurements. Further studies are certainly needed.

Another potential influencing factor relating to robotic technology is the degree of input target resolution [21]. The use of a calliper technique is based on a previously published method of direct calliper measurement, which has the advantage of directly measuring the resection thickness [5]. Challenges include the presence of osteophytes and soft tissue, which should be cleared and may cause potential variations in measurement technique, but despite this, we found a very good interobserver agreement for the measurement values of the bone resections. Another challenge is how to account for the kerf thickness of the blade, which was 1.27 mm in the current study. A method described to account for the kerf thickness of the blade and verify the resected bone thickness is to stack the resected bone on top of the saw blade and then perform the calliper measurement [4]. Our reason for not employing this method is to make our findings comparable to those of previous studies. We found that our average measurement values did not go up to the value of the kerf thickness, but this may explain the standard deviations we found in our results.

Our findings build on the evidence from previous investigations studying patients using robotic technology and the use of the calliper for Kinematic Alignment (KA). When using the Stryker MAKO robotic arm‐assisted technology for TKA, Li et al. 2022 in a single surgeon study with 36 cases, found the femoral distal medial resection to be 0.4 mm ± 0.6 mm, distal lateral femur at 0.5 mm ± 0.7 mm, posterior medial femur at 0.6 mm ± 0.8 mm and posterior lateral femur at 0.7 mm ± 0.8 mm, proximal medial tibia at 0.6 mm ± 0.8 mm and proximal lateral tibia at 0.6 mm ± 0.8 mm [22]. However, the results using robotic surgery can, however, be less accurate than using calliper KA TKA with manual instruments and in the hands of experienced surgeons. In a study of 203 cases, it was found that the distal femur resection can be as low as medial = 0.0 mm ± 0.4 mm, distal femur lateral = 0.0 mm ± 0.5 mm, posterior femur medial = −0.1 mm ± 0.5 mm and posterior femur lateral = −0.1 mm ± 0.5 mm. This is in comparison to data from less experienced surgeons (*n* = 58), which reported distal femur medial = 0.3 mm ± 0.5 mm, distal femur lateral = 0.4 mm ± 0.6 mm, posterior femur medial = − 0.2 mm ± 0.5 mm and posterior femur lateral = −0.4 mm ± 0.5 mm [23]. We have summarised the findings of the current study for all cases and for both varus and valgus knees, with values going down to 0 mm planned versus actual for the femoral cuts (Tables 2, 3a and 3b). Therefore, to consistently achieve ± 0.5 mm of accuracy in keeping with the target for KA, there is a role and little requirement for further optimisation of robotic technology for this alignment strategy; however, one has to consider the associated learning curve and outcomes. We have previously investigated time efficiency and recorded that up to 70 cases are required to achieve similar operative time to the manual technique using mechanical alignment, and that patient satisfaction and pain levels can be better at the 6‐month follow‐up stage [24]. A recent development is the investigation of robotic technology, for example, the NAVIO system for an alignment strategy such as KA [25]. With the aid of 3‐point cartilage thickness measurement and the use of a digital calliper, the authors demonstrated promising results in 142 TKAs with significant correction of the hip‐knee‐ankle (HKA) angle, joint line convergence angle (JLCA) and the mechanical medial proximal tibial angle (mMPTA), but not reporting on the actual bone resection thickness due to the use of a burr instead of a saw [25]. In terms of optimisation of implant position, better restoration of joint line and posterior condylar offset can also be achieved using robotic technology such as the NAVIO and ROSA Knee Systems [26, 27, 28]. In the current study, we did not report on the accuracy of the cut angle as previously described in the cadaveric studies that validated the angle of the cut by the ROSA Knee System using additional navigation systems, which have limitations such as equipment accuracy and evaluation of femoral torsion [5, 11]. If future studies are to include angular measurements in patients, the challenges of this can be the inaccurate estimation of the native femoral and tibial mechanical axes better estimated using exposed bone. The assessment of the degree of cartilage wear on the table and using photographs can be subjective, and there can be a variation of 2–3 mm in the native cartilage thickness [29].

One of the limitations of this study may be discordance between the point of registration and the point of the calliper measurement due to uneven cartilage wear. Another limitation is difficulty achieving accurate registration by the pointer or potential cartilage penetration for difficult‐to‐reach areas such as the posterior condyles if there is, for example, an inability to achieve adequate flexion. We also have to stress that there may be a difference when compared to robotic systems that measure using the imageless technique and those based on preoperative radiographic evaluation and comparative studies are required to further assess the accuracy between the systems for planned and actual measurements in patients with varus and valgus deformity. Although the overall sample size was justified after performing the power analysis, the subgrouping of varus and valgus resulted in undersizing of the valgus group. We acknowledge that the non‐significance found in the four valgus cases, could be a type II error.

In conclusion, the current study in 44 cases found that the use of the ROSA robotic TKA system can achieve accurate bone cuts, comparable to the findings of previous cadaveric studies also utilising the ROSA robotic TKA system (Zimmer Biomet). This study contributes to the overall aim of building evidence for individual robotic systems. Further study should include measuring the degree of accuracy in achieving the planned angular resection in patients and achieving the desired alignment depending on the strategy employed and assessing the time efficiency, learning curves and long‐term outcomes.

## Author Contribution

Z.G. contributed to concept and design; acquisition, analysis, or interpretation of data; drafting of the manuscript; critical review of the manuscript for important intellectual content; and supervision. E.K. contributed to concept and design, acquisition, analysis, or interpretation of data, and drafting of the manuscript. G.D. contributed to the acquisition, analysis or interpretation of data. N.M. contributed to the acquisition, analysis or interpretation of data. A.M. contributed to concept and design, critical review of the manuscript for important intellectual content, and supervision. E.T. contributed to concept and design, critical review of the manuscript for important intellectual content, and supervision.

## Ethics Statement

Our Institutional Review Board (IRB) approval was obtained for the study from the faculty of health sciences, medical department, Bioethics and Ethics committee, Aristotle University, Thessaloniki, Greece.

## Consent

Informed consent was obtained from all the individual participants included in the study.

## Conflicts of Interest

E.Tsiridis is on the editorial board of The Journal of Arthroplasty.

## Data Availability

The data that support the findings of this study are available from the corresponding author upon reasonable request.
